# C16-Ceramide Analog Combined with Pc 4 Photodynamic Therapy Evokes Enhanced Total Ceramide Accumulation, Promotion of DEVDase Activation in the Absence of Apoptosis, and Augmented Overall Cell Killing

**DOI:** 10.1155/2011/713867

**Published:** 2010-12-14

**Authors:** Duska Separovic, Ziad H. Saad, Ethan A. Edwin, Jacek Bielawski, Jason S. Pierce, Eric Van Buren, Alicja Bielawska

**Affiliations:** ^1^Department of Pharmaceutical Sciences, Eugene Applebaum College of Pharmacy and Health Sciences, Wayne State University, 259 Mack Avenue, Detroit, MI 48201, USA; ^2^Karmanos Cancer Institute, 4100 John R, Wayne State University, Detroit, MI 48201, USA; ^3^Department of Biochemistry and Molecular Biology, Medical University of South Carolina, 173 Ashley Avenue, Charleston, SC 29425, USA

## Abstract

Because of the failure of single modality approaches, combination therapy for cancer treatment is a promising alternative. Sphingolipid analogs, with or without anticancer drugs, can improve tumor response. C16-pyridinium ceramide analog LCL30, was used in combination with photodynamic therapy (PDT), an anticancer treatment modality, to test the hypothesis that the combined treatment will trigger changes in the sphingolipid profile and promote cell death. Using SCCVII mouse squamous carcinoma cells, and the silicone phthalocyanine Pc 4 for PDT, we showed that combining PDT with LCL30 (PDT/LCL30) was more effective than individual treatments in raising global ceramide levels, as well as in reducing dihydrosphingosine levels. Unlike LCL30, PDT, alone or combined, increased total dihydroceramide levels. Sphingosine levels were unaffected by LCL30, but were abolished after PDT or the combination. LCL30-triggered rise in sphingosine-1-phosphate was reversed post-PDT or the combination. DEVDase activation was evoked after PDT or LCL30, and was promoted post- PDT/LCL30. Neither mitochondrial depolarization nor apoptosis were observed after any of the treatments. Notably, treatment with the combination resulted in augmented overall cell killing. Our data demonstrate that treatment with PDT/LCL30 leads to enhanced global ceramide levels and DEVDase activation in the absence of apoptosis, and promotion of total cell killing.

## 1. Introduction

Sphingolipids (SLs) are structural components of cell membranes, as well as important players in a variety of biological functions [1]. Ceramide and sphingosine have been associated with proapoptotic and tumor-suppressor functions, whereas sphingosine-1-phosphate (S1P) appears to have prosurvival and tumor-promoting effects [2, 3]. 

Targeting SLs has been used for new anticancer drug design [4, 5]. Exposure of chemoresistant MCF-7 cells to short-chain ceramide analogs, for example, 4,6-dieneceramide, leads to the accumulation of endogenous ceramide, apoptosis, and inhibition of clonogenic survival [6–8]. Cationic ceramide conjugates with pyridinium salts (CCPS analogs) were synthesized that target mitochondria and with improved physicochemical properties [9]. Mitochondrial targeting is important for cancer treatment because cancer cells tend to have mitochondria with more negative mitochondrial membrane potential [10]. These novel ceramide analogs (C2-C16 CCPS) are potent anticancer agents, alone or in combination with standard chemotherapy [9, 11–14]. Exposure of MCF-7 cells to the short-chain homolog LCL29 (C6-CCPS) targets mitochondria to trigger cell killing [15]. Similarly, LCL124, the (2S,3S) isomer of LCL29, alone or in combination with gemcitabine, had potent anticancer activity against human head and neck squamous cell carcinomas in vitro and in vivo [12]. Long-chain ceramide analog LCL30 (C16-CCPS) was shown to target mitochondria and to trigger cell death in SW403 human colon carcinoma cells [13]. In addition, LCL30 was an effective anticancer agent in a colorectal cancer mouse model [14].

Photodynamic therapy (PDT) is a treatment modality for effective abolishment of malignancies including head and neck cancers. In PDT, a light-absorbing agent (photosensitizer) is activated by highly-focused laser light to trigger oxidative stress and destruction of a cellular target [16, 17]. PDT alone, however, can be ineffective with some tumors [18, 19]. To overcome inefficiency of PDT itself to eradicate tumors, combined treatments become a necessary option. Using LCL29 [9, 20] in combination with Photofrin-PDT, we have shown moderately improved in vivo response of mouse SCCVII squamous carcinomas [21]. 

**Figure 1:**
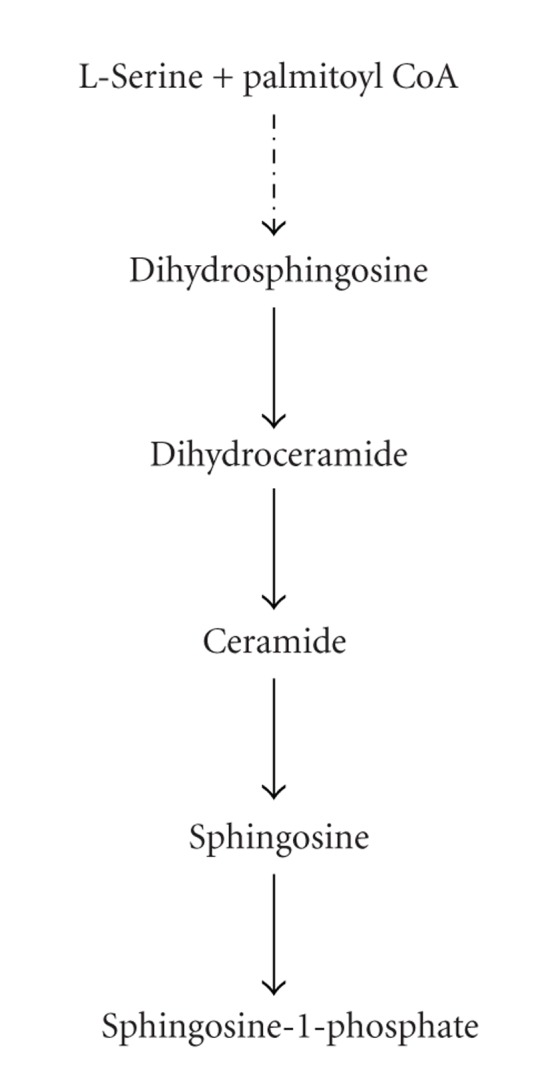
De novo ceramide metabolism.

Here, we used for the first time LCL30 in combination with PDT to determine their effects on endogenous SLs, apoptosis, and clonogenic survival in SCCVII mouse squamous carcinoma cells (SCCVII cells). The model was chosen in order to use the results of this study in our future in vivo work in syngeneic mouse SCCVII squamous carcinomas, a recognized mouse model for human head and neck cancers [22], since intact immune system is a key to PDT therapeutic success [16, 17]. For PDT, silicon phthalocyanine Pc 4, a photosensitizer with physicochemical properties superior to Photofrin [23, 24], was chosen for these investigations. SLs that were analyzed by mass spectrometry (MS) are shown in Figure 1 as part of, or relative to the de novo ceramide pathway. Others [25–30] and we [21, 31–35] have shown that the de novo ceramide pathway modulates response to anticancer drugs, including PDT. For example, we have shown in Jurkat cells that silencing of ceramide-utilizing enzyme sphingomyelin synthase leads to enhanced ceramide and dihydroceramide accumulation with concomitant promotion of apoptosis [32]. Combining PDT with the anticancer LCL30 is expected to promote tumor response to PDT and, therefore, improve clinical PDT.

## 2. Materials and Methods

### 2.1. Materials

D-erythro-2-*N*-[16′-(1′′-pyridinium)-hexadecanoyl]-sphingosine bromide (LCL30) was synthesized in the Lipidomics Shared Resource of the Medical University of South Carolina [9]. The phthalocyanine photosensitizer Pc 4, HOSiPcOSi(CH3)2(CH2)3N(CH3)2, was supplied by Dr. Malcolm E. Kenney (Department of Chemistry, Case Western Reserve University). RPMI medium and serum were from Invitrogen and Hyclone, respectively.

### 2.2. Cell Culture and Treatments

SCCVII cells were grown in RPMI medium (Invitrogen) containing 10% fetal bovine serum (Hyclone) 100 units/mL penicillin and 100 *μ*g/mL streptomycin. Cells were maintained at 37°C in a 5% CO_2_ atmosphere and were treated in the growth medium. For PDT experiments, after overnight incubation with Pc 4 at 37°C, cells were irradiated with red light (2 milliwatts/cm^2^; *λ*_max_ ~ 670 nm) using a light-emitting diode array light source (EFOS) at the fluence of 200 mJ/cm^2^ at room temperature. Following PDT, cells were incubated at 37°C for 2 hours. For the combination, LCL30 (1 *μ*M) was added to Pc 4-pretreated cell cultures 2 hours prior to irradiation. For cells treated with LCL30 alone, the incubation time was 4 hours. Following treatments, cells were collected on ice and processed for various analyses. For MS analysis, cells were washed twice with cold phosphate-buffered saline (PBS), resuspended in the mixture of ethyl acetate/methanol (1 : 1, v/v), dried under nitrogen, and shipped overnight on dry ice to the Lipidomics Shared Resource (Charleston, SC) for further processing.

### 2.3. Electrospray Ionization/Double MS Analysis

Following extraction, SLs were separated by high-performance liquid chromatography, introduced to electrospray ionization source, and then analyzed by double MS using TSQ 7000 triple quadrupole mass spectrometer (Thermo-Fisher Scientific) as described previously [32].

### 2.4. Mitochondrial Membrane Depolarization Measurement

As we showed previously [36], the lipophilic cationic dye JC-1 (5,5′,6,6′-tetrachloro-1,1′3,3′-tetraethylbenzimidazolylcarbocyanine iodide; Molecular Probes) was used to determine mitochondrial membrane potential by flow cytometry. JC-1 forms aggregates in normal mitochondria with a high negative membrane potential that emit a red fluorescence (590 nm). In mitochondria with low membrane potential (depolarized), the dye forms monomers in the cytosol that emit a green fluorescence (527 nm) [37]. Following treatments, cells were collected, washed in PBS, resuspended in JC-1-containing medium (2 *μ*M final concentration), and incubated at 37°C for 15 minutes. The samples were centrifuged, resuspended in growth medium, and analyzed by flow cytometry using BD LSR II flow cytometer (BD Biosciences). As a positive control for mitochondrial depolarization and apoptosis, cells were treated with camptothecin (5 *μ*M) overnight and processed for flow cytometry.

### 2.5. Apoptosis Detection

As we showed previously [38], to detect apoptosis, the exposure of phosphatidylserine in the outer leaflet of the cell membrane was measured using Annexin V, a protein which binds with high affinity to negatively charged phosphatidylserine in the presence of calcium. As apoptosis progresses, cell membrane integrity is lost, and this can be detected using DNA-binding fluorescent dye propidium iodide (PI, red fluorescence). By attaching Annexin V to the fluorescein isothiocyanate (FITC; green fluorescence), one can discriminate between intact cells (Annexin V negative, PI negative), early apoptotic (Annexin V positive, PI negative), and late apoptotic or necrotic cells (Annexin V positive, PI positive). The kit was purchased from BD Pharmingen and the flow cytometry protocol was followed, as described by the manufacturer. 

### 2.6. DEVDase (Caspase-3-Like) Activity Assay

As described previously [34], the activity of the apoptotic marker DEVDase was determined in cytosol by an assay based on the enzyme's ability to cleave the fluorogenic derivative 7-amino-4-methylcoumarin (AMC; Biomol) of the tetrapeptide substrate N-acetyl-Asp-Glu-Val-Asp (DEVD). The released fluorescence of the cleaved DEVD substrate was measured in F-2500 Hitachi spectrofluorometer (380 nm excitation and 460 nm emission).

### 2.7. Clonogenic Assay

Cell viability was assessed using clonogenic assay. The protocol that we described previously [38, 39] was modified using preplating approach, which allows keeping the treatments in the medium during colony formation. Following addition of Pc 4 or LCL30, cells were seeded in the growth medium, preincubated overnight, and irradiated. After eight days of growth at 37°C, colonies (≥50 cells) were stained with crystal violet (0.1%) and counted. Plating efficiency was 76% (*n* = 81).

### 2.8. Statistical Analysis

Statistical differences were determined using Student's *t*-test (*P* < .05).

## 3. Results and Discussion

### 3.1. LCL30 Is Taken up by SCCVII Cells

**Figure 2:**
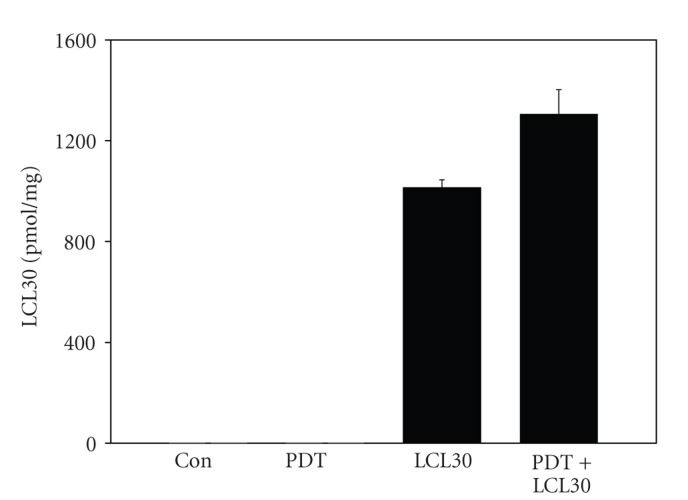
LCL30 is taken up by SCCVII cells treated with LCL30 or (PDT+LCL30). (PDT+LCL30): following overnight exposure to Pc 4 (250 nM), cells were treated with LCL30 (1 *μ*M) for 2 hours, irradiated with red light (200 mJ/cm^2^), and incubated for additional 2 hours. For cells treated with LCL30 alone, the incubation time was 4 hours. Following incubations, cells were collected and processed for MS. The LCL30 levels are expressed as actual values (pmol/mg) and are shown as average ± SEM from four independent determinations.

We showed rapid uptake of LCL30 by MCF-7 cells [9]. Here, we determined uptake of LCL30 by SCCVII cells using MS. Following a four-hour incubation of cells with LCL30 (1 *μ*M), cellular levels of LCL30 were 260 ± 4 pmol/10^6^ cells, corresponding to approximately 5% of the concentration applied. After PDT (250 nM Pc 4 + 200 mJ/cm^2^) + LCL30 (1 *μ*M), cellular levels of LCL30 were increased by 29% (Figure 2). The increase was marginally significant (*P* < .051).

### 3.2. PDT/LCL30 Is More Effective Than Individual Treatments in Raising Global Levels of Ceramides

**Figure 3:**
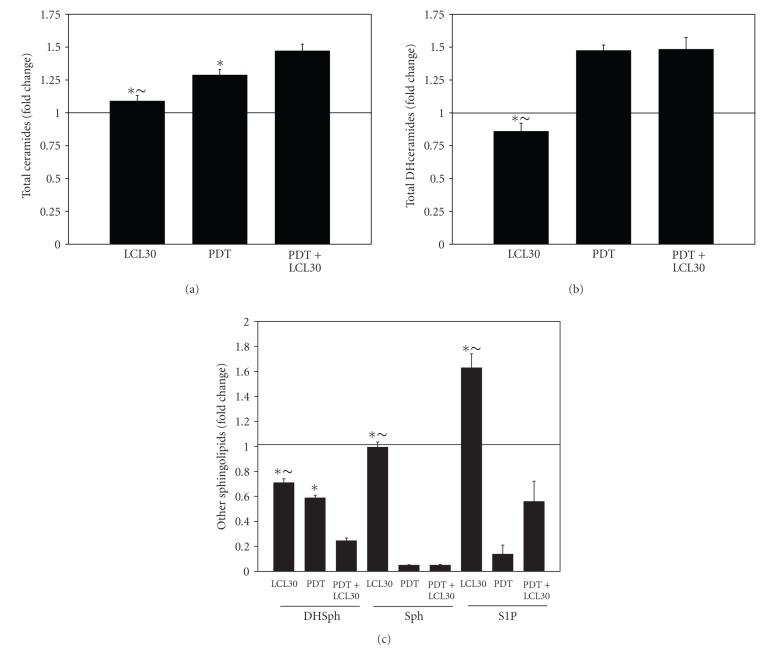
The effects of treatments on the SL profile. (a)–(c) Total ceramides, DHceramides, and other SLs, respectively. The data are expressed as fold changes, that is, ratios of treated versus controls (Pc 4 alone and untreated controls) and are shown as average ± SEM from four independent determinations. For other details, refer to Figure 2 legend. The significance (*P* < .05) is shown as follows: * indicates the significant difference between an individual treatment, LCL30 or PDT, and the combination (PDT+LCL30); ~ indicates the significant difference between LCL30 and PDT. DHSph, dihydrosphingosine; Sph, sphingosine.

We have already demonstrated that PDT has signature effects on the SL profile in cancer cell lines and in vivo [21, 32, 39]. We [9] and others [14] have also shown that LCL30 triggers changes in endogenous ceramides and S1P in human cancer cell lines. In the present study, we used MS to determine the effects of the combination, as well as of the individual treatments, on the SL profile in SCCVII cells. The effects of treatments on ceramides, individual and global, are shown in Table 1 and Figure 3(a). Only C20:1-ceramide was elevated after all treatments. Globally, there was 1.29- and 1.47-fold increase in ceramide levels after PDT and PDT/LCL30, respectively. LCL30 had no effect on total ceramides. The differences between individual treatments, LCL30 or PDT, and the combination, as well as between individual treatments themselves, were significant (*P* < .05).

The effects of treatments on the levels of dihydroceramides (DHceramides), individual and global, are depicted in Table 1 and Figure 3(b). Maximal increases were observed for C22- and C22:1-DHceramide after PDT and PDT/LCL30, respectively. There was no significant effect of LCL30 on total DHceramides. In contrast, PDT increased total DHceramides 1.48-fold, and the effect was maintained after the combination. 

The effects of treatments on other SLs are shown in Table 1 and Figure 3(c). The levels of dihydrosphingosine (DHsphingosine), a metabolic precursor of DHceramide, were reduced by 29, 41, and 75% after LCL30, PDT, and PDT/LCL30, respectively. The differences between treatments were significant (*P* < .05). Thus, PDT/LCL30 was more effective than individual treatments in reducing DHsphingosine levels. Sphingosine levels were abolished after PDT or the combination. LCL30 had no effect on sphingosine accumulation. In contrast, LCL30 triggered a marked 1.63-fold rise in S1P levels. PDT reduced S1P levels by 86%, and in combination with LCL30, by 44%.

### 3.3. PDT, with or without LCL30, Has No Effect on Mitochondrial Membrane Potential

**Figure 4:**
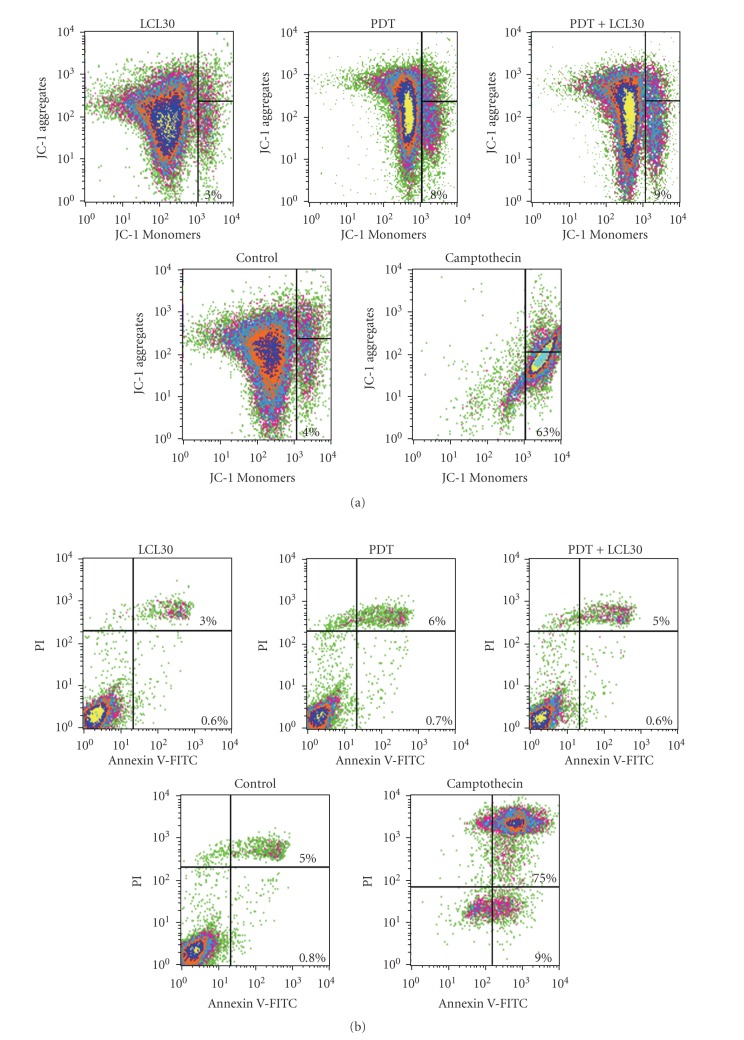
PDT, with or without LCL30, has no effect on mitochondrial membrane potential or apoptosis. Following incubations, cells were collected and processed for flow cytometry. JC-1 and Annexin V/PI staining were used to detect mitochondrial membrane potential (a) and apoptosis (b), respectively. See Section 2 for other details. (a) Percentage of cells with depolarized mitochondria is shown in lower right dot plot. (b) Percentage of Annexin V (+)/PI (−) and Annexin V (+)/PI (+) is shown in lower right and upper right dot plot, respectively. The representative data for PDT, with or without LCL30, from two independent experiments are shown.

We have shown that PDT combined with C16-ceramide enhances mitochondrial depolarization in Jurkat cells undergoing apoptosis [36]. Here, we tested whether PDT/LCL30 triggers the collapse of mitochondrial potential in SCCVII cells. As depicted in Figure 4(a), neither individual treatments nor the combination had any effect on mitochondrial membrane potential. In contrast, overnight treatment with camptothecin (5 *μ*M) led to the appearance of 63% cells with depolarized mitochondria.

### 3.4. PDT, with or without LCL30, Has No Effect on Apoptosis

PDT is a potent apoptotic inducer [40]. Surprisingly, in the presence of PDT, with or without LCL30, no apoptosis was detected in SCCVII cells using either Annexin V/PI staining (Figure 4(b)) or TUNEL assay (not shown). However, overnight treatment with camptothecin (5 *μ*M) led to the appearance of 9% and 75% cells that were Annexin V (+)/PI (−) and Annexin V (+)/PI (+), respectively. Thus, unlike other treatments, camptothecin triggers early and late apoptosis/necrosis.

### 3.5. PDT/LCL30 Enhances DEVDase Activation

**Figure 5:**
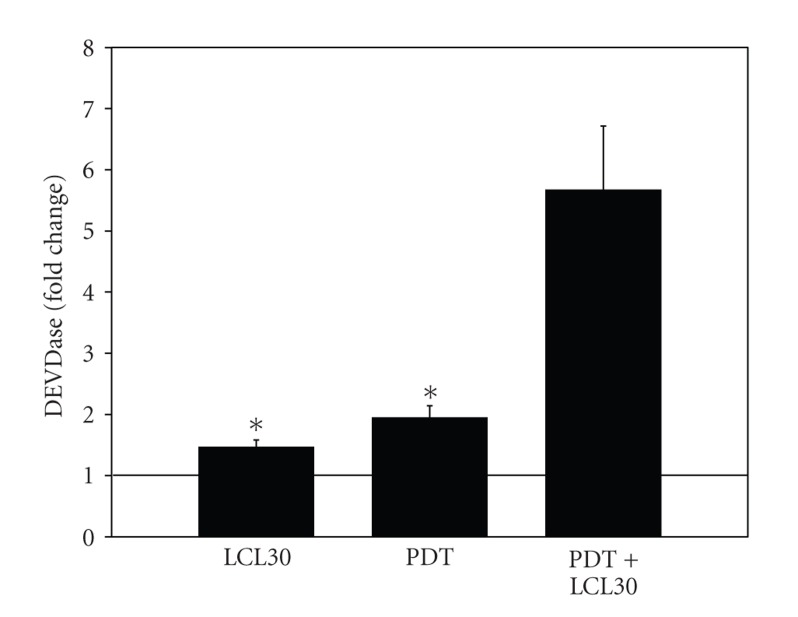
DEVDase activation is enhanced after (PDT+LCL30). Following incubations (see Figure 2 legend), cells were collected, cell lysates were prepared, and DEVDase activity was measured using DEVD-AMC as the fluorogenic substrate. The data are expressed as fold changes, that is, ratios of treated versus untreated controls and are shown as average ± SEM from four to six independent determinations. The significance (*P* < .05) is shown by an asterisk indicating significant difference between an individual treatment, LCL30 or PDT, and the combination (PDT+LCL30).

Although LCL30 was reported not to induce apoptosis [14], PDT is an effective apoptotic inducer [41]. We used DEVDase assay to assess the activity of caspase-3 as an apoptotic marker. DEVDase was activated 1.47- and 1.95-fold after LCL30 and PDT, respectively (Figure 5). After treatment of SCCVII cells with the combination, a 5.68-fold increase in DEVDase activity was recorded. Therefore, the combined treatment augments the enzyme activity.

Previously, we showed that enhanced ceramide accumulation is associated with increased activation of DEVDase after PDT [32, 35]. We also showed that DEVDase activation is accompanied by mitochondrial depolarization and/or apoptosis post-PDT [33–36, 42, 43]. The present study shows DEVDase activation in the absence of mitochondrial depolarization and apoptosis after PDT, with or without LCL30. Caspases, including caspase-3, have been associated with nonapoptotic functions, for example, survival [44–46]. Curiously, there was positive correlation between DEVDase activation and the levels of prosurvival S1P after LCL30. In addition, there was negative correlation between the proapoptotic DHsphingosine [33] and DEVDase activation. The levels of proapoptotic sphingosine [47] were abolished after PDT or the combination with concomitant activation of DEVDase. The role of DEVDase activation in the absence of apoptosis in response to PDT, with or without LCL30, remains to be established.

### 3.6. PDT/LCL30 Augments Cell Killing

**Figure 6:**
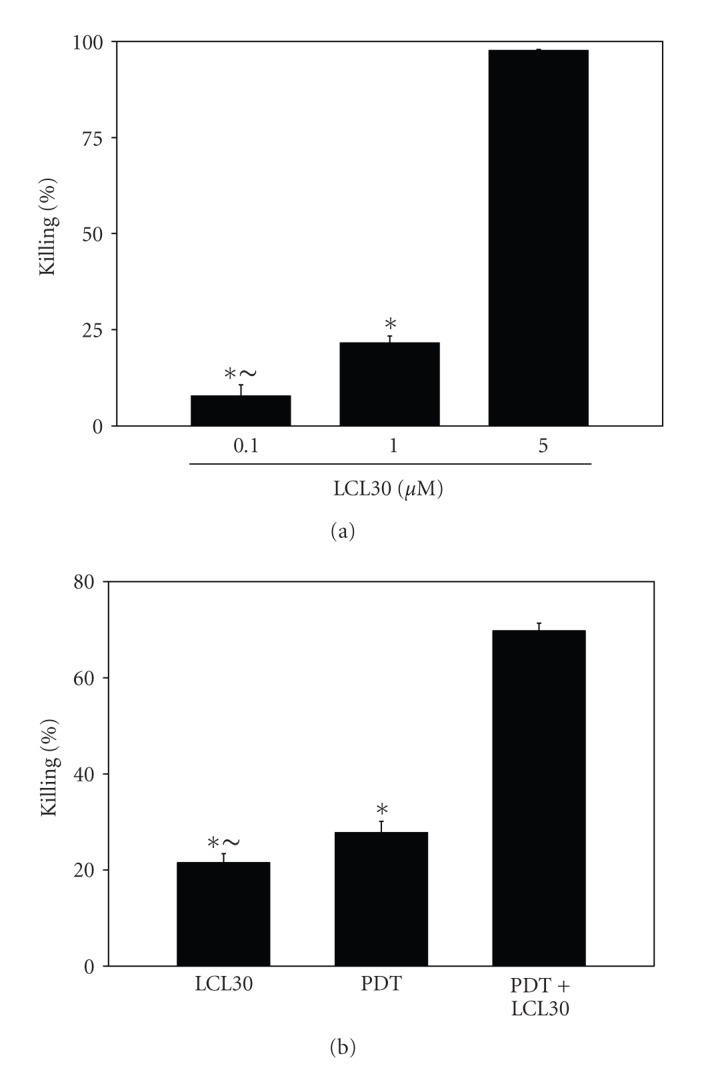
Overall cell killing is promoted after (PDT+LCL30). Following the addition of Pc 4 or LCL30, cells were seeded in the growth medium, preincubated overnight, and irradiated with red light (200 mJ/cm^2^). After eight days of growth at 37°C, colonies (≥50 cells) were stained with crystal violet (0.1%) and counted. The data are expressed as the percentage of killing and are shown as average ± SEM from six to 26 independent determinations. (a) Dose-dependent increase in cell killing after LCL30. The significance (*P* < .05) is shown as follows: * indicates the significant difference between lower doses (0.1 or 1 *μ*M) and the highest LCL 30 dose (5 *μ*M); ~ indicates the significant difference between 0.1 and 1 *μ*M LCL30. (b) Total cell killing is augmented after (PDT + 1 *μ*M LCL30). The significance (*P* < .05) is shown as follows: * indicates the significant difference between an individual treatment, LCL30 or PDT, and the combination (PDT+LCL30); ~ indicates the significant difference between LCL30 and PDT.

To determine the effect of LCL30 on overall cell killing, clonogenic assay was employed. As shown in Figure 6(a), treatment of SCCVII cells with 0.1, 1 and 5 *μ*M LCL30 led to 7.8, 21.5 and 97.7% cell killing, respectively. The differences between each dose were significant (*P* < .05), supporting a dose-dependent cell killing after LCL30. 

To test for potential enhancement of cell killing by the combination, LDs < 30 were chosen for each treatment. When SCCVII cells were treated with LCL30 (1 *μ*M) and PDT (250 nM Pc 4 + 200 mJ/cm^2^), 69.8% of the overall cell killing was observed (Figure 6(b)). The combination led to cell killing that was greater than that by each treatment alone (*P* < .05). 

Taken together, the present study demonstrates for the first time that (i) LCL30 is taken up by SCCVII cells; (ii) cellular total ceramides are enhanced after PDT/LCL30; (iii) the activity of DEVDase is upregulated in the absence of mitochondrial depolarization and apoptosis post-PDT/LCL30; (iv) total cell killing is augmented after the combination. Our findings warrant the use of other apoptotic markers besides DEVDase to verify the triggering of apoptosis, and in vivo testing of PDT/LCL30 in view of translational potential of the combination.

## Figures and Tables

**Table 1:** The effect of LCL30, PDT, and PDT + LCL30 on the SL profile in SCCVII cells.

	LCL30	PDT	PDT + LCL30
	Sphingolipids^a^		
C14-Cer	**1.35**	0.90	**1.33**
C16-Cer	1.02	1.13	**1.53**
C18-Cer	1.18	**1.52**	**1.70**
C18:1-Cer	1.12	1.19	**1.48**
C20-Cer	1.13	**1.53**	**1.69**
C20:1-Cer	**1.64**	**1.65**	**2.06**
C22-Cer	0.82	**1.43**	**1.42**
C22:1-Cer	**1.23**	1.11	**1.41**
C24-Cer	**0.82**	1.07	1.19
C24:1-Cer	1.09	1.09	**1.37**
C26-Cer	0.94	**1.39**	1.05
C26:1-Cer	0.77	**1.46**	**1.46**
C14-DHCer	0.71	1.60	1.64
C16-DHCer	**0.72**	**1.52**	1.22
C18-DHCer	0.67	1.25	1.00
C20-DHCer	0.96	**1.30**	1.40
C22-DHCer	0.97	**1.90**	**1.70**
C22:1-DHCer	1.20	**1.73**	**2.28**
C24-DHCer	**0.67**	1.16	1.14
C24:1-DHCer	0.99	**1.36**	**1.51**
DHSph	**0.71**	**0.59**	**0.25**
Sph	1.00	**0.05**	**0.05**
S1P	**1.63**	**0.14**	0.56

^a^The data are shown as ratios compared to controls (untreated and Pc 4 alone). Significant values at *P* < .05 are shown in bold. C14-Cer, C14-ceramide, and so forth; C14-DHCer, C14-dihydroceramide, and so forth; DHSph, dihydrosphingosine; Sph, sphingosine.
